# Exposure to *Toxoplasma gondii* in Asian Elephants (*Elephas maximus indicus*) in Thailand

**DOI:** 10.3390/pathogens11010002

**Published:** 2021-12-21

**Authors:** Ruenruetai Udonsom, Yoshifumi Nishikawa, Ragab M. Fereig, Thitirat Topisit, Natchakorn Kulkaweewut, Supitcha Chanamrung, Charoonluk Jirapattharasate

**Affiliations:** 1 Department of Protozoology, Faculty of Tropical Medicine, Mahidol University, 420/6 Ratchawithi Road, Ratchathewi, Bangkok 10400, Thailand; Ruenruetai.udo@mahidol.ac.th; 2 National Research Center for Protozoan Diseases, Obihiro University of Agriculture and Veterinary Medicine, Obihiro 080-8555, Hokkaido, Japan; Nisikawa@obihiro.ac.jp (Y.N.); ragabfereig2018@gmail.com (R.M.F.); 3 Department of Animal Medicine, Faculty of Veterinary Medicine, South Valley University, Qena 83523, Egypt; 4 Department of Pre-clinic and Applied Animal Science, Faculty of Veterinary Science, Mahidol University, 999 Phuthamonthon sai 4 Rd, Salaya, Nakhon Pathom 73170, Thailand; Thitirat.top@student.mahidol.edu (T.T.); Natchakorn.kul@student.mahidol.edu (N.K.); Supitcha.cha@student.mahidol.edu (S.C.)

**Keywords:** elephants, indirect enzyme-linked immunosorbent assay, latex agglutination test, recombinant GRA8 protein, *T. gondii* lysate antigens, serology, Thailand

## Abstract

*Toxoplasma gondii* is the causative agent of toxoplasmosis in humans and various animal species worldwide. In Thailand, seroprevalence studies on *T. gondii* have focused on domestic animals, and information on infections in Asian elephants (*Elephas maximus indicus*) is scarce. This study was conducted to determine the seroprevalence of *T. gondii* infection in archival sera collected from 268 elephants living in Thailand. The serum samples were analyzed for anti-*T. gondii* immunoglobulin G antibodies using the latex agglutination test (LAT) and indirect enzyme-linked immunosorbent assay (iELISA) based on *T. gondii* lysate antigen (TLA-iELISA) and recombinant *T. gondii* dense granular antigen 8 protein (TgGRA8-iELISA). The prevalence of antibodies against *T. gondii* was 45.1% (121/268), 40.7% (109/268), and 44.4% (119/268) using LAT, TLA-iELISA, and TgGRA8-iELISA, respectively. Young elephants had a higher seropositivity rate than elephants aged >40 years (odds ratio = 6.6; *p* < 0.001; 95% confidence interval: 2.9–15.4). When LAT was used as the reference, TLA-iELISA and TgGRA8-iELISA showed a substantial (κ = 0.69) and moderate (κ = 0.42) agreement, respectively. Although our findings suggest the widespread exposure of Asian elephants to *T. gondii* in Thailand, the source of infection was not investigated. Therefore, investigation of the predisposing factors associated with toxoplasmosis is necessary to identify the potential risk factors for infection.

## 1. Introduction

*Toxoplasma gondii* is an obligate apicomplexan parasite that is widely prevalent in most warm-blooded animals, including humans [1,2]. The definitive hosts are domestic and wild felids, which excrete environmentally robust oocysts in their feces [3]. Intermediate hosts are infected via the fecal–oral route through the ingestion of food, water, or soil contaminated with sporulated oocysts, by consuming tissue cysts, or by congenital transmission [4].

*Toxoplasma* sp. infections have been reported in both humans and domestic animals in Thailand. The prevalence of anti-*T. gondii* antibodies in Thai people is 3.1–53.7% [5,6,7,8]. *T. gondii* antibodies have been detected in cats [9,10,11], dogs [9,12], cattle [13,14,15], chickens [16], and goats [17,18]. The seroprevalence in captive wild felids in Thailand was reported as 15.4–42.8% [19,20]. Toxoplasmosis in herbivores including elephants might be caused by ingesting food or water contaminated with sporulated oocysts. Furthermore, the infected herbivorous matter indicates environmental contamination with *T. gondii* oocysts [21]. The infection of the parasite in elephants can be used to monitor disease circulation and infection risks for human or wild populations. However, information about the prevalence of *T. gondii* infection in Asian elephants (*Elephas maximus indicus*) in Thailand is scarce.

Serological assays are essential for the detection of *T. gondii* infection in humans and animals [22]. Various techniques have been employed to screen for specific antibodies against *T. gondii*, such as the modified agglutination test (MAT), latex agglutination test (LAT), and indirect fluorescent antibody test (IFAT). Furthermore, an enzyme-linked immunosorbent assay (ELISA) based on soluble *T. gondii* lysate antigens (TLAs) has been used to detect *T. gondii*-specific antibodies in animals [23,24,25,26]. Recently, recombinant antigenic proteins of *T. gondii* have been widely used for serodiagnosis of toxoplasmosis in various animal species [27]. Several target antigens of *T. gondii* have been evaluated and applied in serological tests, such as the surface antigen, microneme antigen, rhoptry antigen, and dense granule antigen (GRA) tests. Antigens from the GRA group, such as GRA5, GRA6, GRA7, and GRA8, have demonstrated diagnostic potential for the serological detection of animal toxoplasmosis [27]. 

This study aimed to investigate the seroprevalence of *T. gondii* infection in Asian elephants living in Thailand using a commercial test kit and compared the results with those obtained using indirect ELISA (iELISA) based on TLA and a recombinant protein of *T. gondii* dense granular antigen 8 (TgGRA8).

## 2. Results

The seroprevalence of IgG antibodies against *T. gondii* in the elephants in various parts of Thailand is presented in Table 1. Antibodies against *T. gondii* were detected in the sera of 121 (45.1%), 109 (40.7%), and 119 (44.4%) of 268 elephants by LAT, TLA-iELISA, and TgGRA8-iELISA, respectively. The antibody titers determined by LAT were 1:64, 1:128, 1:256, 1:512, 1:1024, and 1:2048 in 22, 36, 21, 27, 8, and 7 elephants, respectively. Significant differences were observed in the seroprevalence of *T. gondii* among the age groups. The prevalence of *T. gondii* infection in elephants aged >40 years was significantly lower than that in those aged 21–40 years (*p* = 0.002) and those aged 1–20 years (*p* < 0.001). There was no significant association between sex and *T. gondii* seroprevalence (Table 2). 

Evaluation of the agreement between the tests revealed that TLA-iELISA had slightly higher sensitivity (78.5%) and specificity (90.4%) than TgGRA8-iELISA (67.7% and 74.8%, respectively). The kappa value between LAT and TLA-iELISA was 0.69 (substantial agreement) and between LAT and TgGRA8-iELISA was 0.42 (moderate agreement) (Table 3).

## 3. Discussion

The seroprevalence of *T. gondii* in domestic and wild animals in Thailand has been investigated in the past [5,6,7,8,9,10,11,12,13,14,15,16,17,18,19,20]. However, there are few reports on *T. gondii* infection in elephants. In this study, the seropositivity rate for *T. gondii* in elephants in captivity was >40%, which was higher than previously reported seropositivity rates in Asian elephants in Thailand, which were reported as 13% [28] and 25.6% [29] by LAT. Because the lifespan of elephants is similar to that of humans, the likelihood of *T. gondii* infection is higher than that in other animals. Moreover, some elephant owners release them to explore and forage for food in nearby forests [30], where they come into contact with plants contaminated with oocysts excreted by wild felids or domestic cats. Thus, a long-term study and risk factor association of *T. gondii* infection in elephants in Thailand is warranted.

We did not find a difference in *T. gondii* seropositivity between female and male elephants, which is consistent with the findings of a previous report that both male and female elephants were equally exposed to *T. gondii* infection [28]. The highest seroprevalence of *T. gondii* was detected in elephants aged 1–20 years and 21–40 years, which is similar to a previous report of high *T. gondii* seroprevalence in adult elephants [28]. However, our results showed a low prevalence of infection in elephants aged >40 years. This could be due to a possible bias and the high number of elephants of unknown age (60/268, 22.4%) in our study cohort. Because no data on congenital toxoplasmosis in elephants are available, further studies on the clinical manifestations of *T. gondii* infection in elephants should be performed.

We used LAT, TLA-iELISA, and TgGRA8-iELISA to detect *T. gondii* infection in elephant sera. Although LAT exhibited low sensitivity compared with MAT, it is widely used as a reference test for the seroprevalence of toxoplasmosis in various animal species, including elephants [28,29,31,32,33]. ELISA is a common serological test that can be easily performed on a large scale, and many commercial kits are available to detect specific immunoglobulins produced in response to *T. gondii* infection. The conventional TLA-iELISA test shows a high degree of agreement with MAT, IFAT, or LAT for detecting antibodies against *T. gondii* in both humans and animals [25,34]. In the present study, TLA-iELISA demonstrated high sensitivity (78.5%) and specificity (90.4%), and the kappa value indicated a substantial agreement with LAT (κ = 0.69) for the detection of *T. gondii* infection in elephant sera. This indicates that TLA-iELISA could be an alternative serological test for *T. gondii* infection in elephants and other wild animals. Recombinant antigens are considered alternative diagnostic antigens to native antigens and have been used to improve the serodiagnosis of *T. gondii* [27]. Recombinant TgGRA8 antigen has been used in ELISA to detect toxoplasmosis in both humans and animals. High sensitivity and specificity for the detection of *T. gondii* have been reported using TgGRA8-iELISA in humans [35], goats [36], and domestic turkeys [37]. This is the first report of using recombinant protein TgGRA8 as a coating antigen to detect antibodies against *T. gondii* in elephants. The kappa value between LAT and TgGRA8-iELISA revealed a moderate agreement (κ = 0.42), with low sensitivity and specificity. Recently, multi-epitope or chimeric antigens have been used as an alternative approach to address the need for standardizing and increasing the sensitivity and specificity of serodiagnostic tests for animal toxoplasmosis [25,26,38]. Therefore, the application of a single recombinant protein or a mixture of recombinant proteins in an ELISA test to diagnose *T. gondii* infection in elephants needs to be developed and evaluated.

The long-term storage of serum affects the stability of several biochemical components and immunoglobulins in human and animal sera [39,40,41]. This could be impacted on the serological tests in our study because most of serum samples were stored for a long time. However, one study revealed that IgG antibodies against *T. gondii* in human sera can be reliably measured for 10 years of storage at −20 °C with no modification of interpretation of toxoplasmosis serologies [41]. To clarify whether long-term storage of such animal sera has an influence on the serological testing of *T. gondii* infection, further study will be necessary.

In conclusion, *T. gondii* infection is fairly common in Asian elephants in Thailand. The prevalence of the infection found in wild animals in Thailand could be of public health and conservation concern. Furthermore, the Asian elephants can be used as sentinels to monitor the potential contamination in the environment with *T. gondii*. However, updated information on the seroprevalence of this infection in elephants and a risk factor analysis should be performed to determine the actual situation and to identify the infection source and possible transmission routes.

## 4. Materials and Methods

### 4.1. Animal Samples

**Figure 1:**
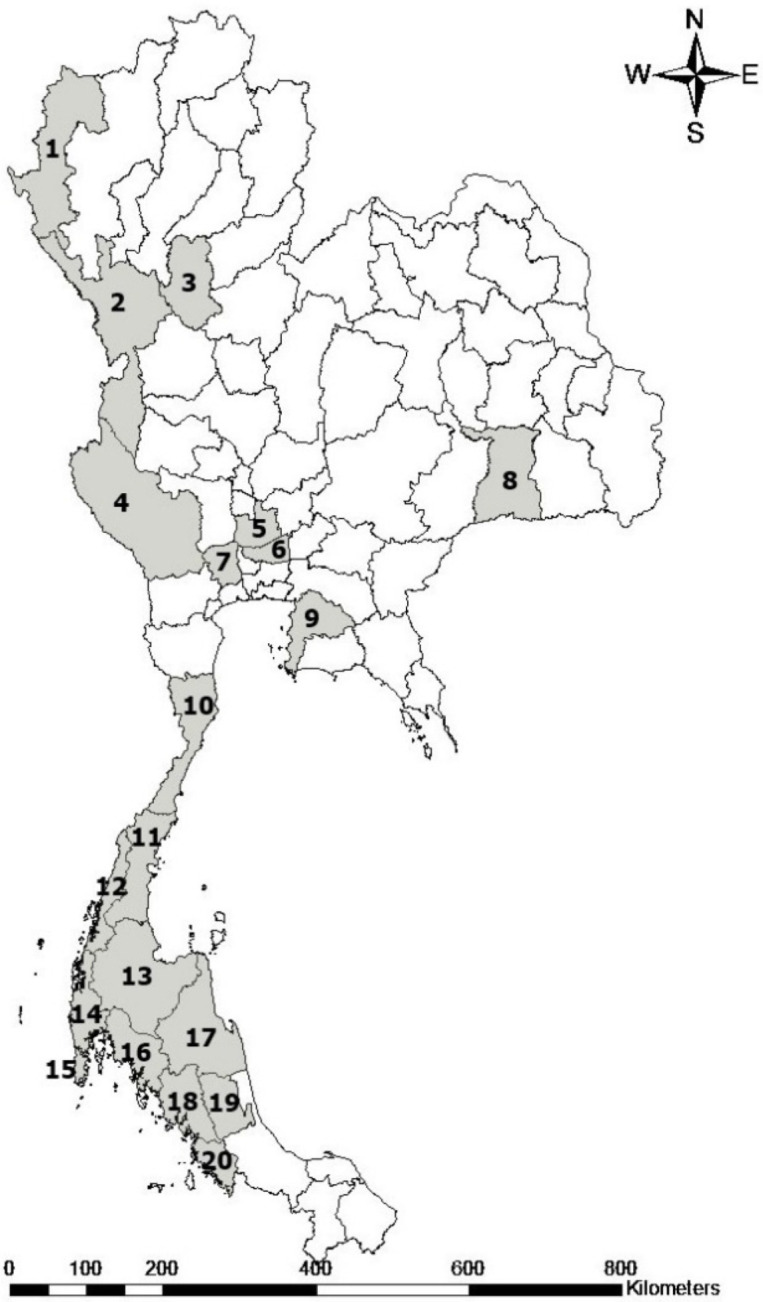
Geographic distribution of the sampling sites in Thailand used in this study. Dark-colored areas with different numbers indicate the investigated provinces. 1. Mae Hong Sorn, 2. Tak, 3. Sukhothai 4. Kanchanaburi, 5. Ayutthaya, 6. Pathum Thani, 7. Nakhon Pathom, 8. Surin, 9. Chonburi, 10. Prachaup Kirikhan, 11. Chumphon, 12. Ranong, 13. Surat Thani, 14. Phangnga, 15. Phuket, 16. Krabi, 17. Nakhon Si Thammarat, 18. Trang, 19. Phatthalung, 20. Satun.

Archival elephant sera (*n* = 268) in this study were obtained from the project on animal DNA fingerprints in a cooperation between the Asian Elephant Foundation of Thailand and the Monitoring and Surveillance Center for Zoonotic Diseases in Wildlife and Exotic Animals, Faulty of Veterinary Science, Mahidol University. The serum samples were collected in various provinces of Thailand from 2009 to 2013 and in 2020 (Figure 1). For this study, the sample was divided into three aliquot parts for the serological tests and frozen at −30 °C. Data were recorded for each animal and included the sampling area, sampling date, sex, and age.

### 4.2. Preparation of T. gondii GRA8 Antigen

Purified recombinant *T. gondii* dense granular antigen 8 (TgGRA8) was prepared as described previously [40]. Briefly, TgGRA8 (582 bp) was amplified using polymerase chain reaction and then ligated into a pET-21a vector using NdeI and XhoI as the cloning sites (General Biosystems, Durham, NC, USA) that were transfected into *Escherichia coli* strain Rosetta (DE3) cells. TgGRA8 protein production was induced using isopropyl-β-D-thiogalactopyranoside to a final concentration of 1 mM with mild shaking at 20 °C overnight. Next, the cells were centrifuged at 4400× *g* for 20 min at 4 °C, and the resultant bacterial pellet was resuspended in 20 mL of prechilled lysis buffer. Then, the cells were disrupted by sonication on ice for 10 min. The total proteins in the soluble fraction were subjected to affinity purification using an anti-DYKDDDDK G1 affinity resin (GenScript, Piscataway, NJ, USA) according to the manufacturer’s protocols. The purity and quantity of the proteins were checked using sodium dodecyl sulfate-polyacrylamide gel electrophoresis analysis. The protein concentration was measured using the BSA assay (Pierce Biotechnology, Inc., Rockford, IL, USA).

### 4.3. Preparation of Toxoplasma Lysate Antigen

Tachyzoites of the *T. gondii* RH strain were cultivated in a monolayer of Vero cells (African green monkey kidney cells) with Dulbecco’s Modified Eagle’s Medium (GIBCO, Grand Island, NY, USA) supplemented with 10% heat-inactivated fetal bovine serum. The pellets were harvested and washed twice in phosphate-buffered saline (PBS), followed by three freeze–thaw cycles, based on the standard procedure. The protein concentration in the TLA preparation was measured using Bradford reagent (Sigma-Aldrich, St. Louis, MO, USA) according to the manufacturer’s recommendations, and the proteins were stored at −20 °C until use.

### 4.4. Latex Agglutination Test

We tested the elephant sera for *T. gondii* infection using a MAST Toxoreagent LAT (Mast Group, Liverpool, UK), according to the manufacturer’s instructions. Samples were considered positive when agglutination was observed at a dilution of ≥1:64 [29].

### 4.5. IgG Indirect ELISA (iELISA)

iELISA was performed to detect *T. gondii* infection according to a previously described procedure [39], with slight modifications. Optimal dilutions were established using checkerboard titrations with dilutions of coating antigen and sera. The ELISA plates (Nunc, Roskilde, Denmark) were coated with 0.1 mL of TLA or TgGRA8 (1 μg/mL) diluted in a coating buffer (50 mM carbonate, pH 9.6) and were incubated at 4 °C overnight. Then, the plates were rinsed five times with PBS containing 0.05% Tween 20 (PBS-T), and nonspecific immune sites were blocked with 5% PBS-skimmed milk (PBS-SM) for 1 h at 37 °C. After washing with PBS-T, the control and sample sera were diluted to 1:100 in PBS-SM, and 100 μL of this mixture was added to each well in duplicate. After incubation at 37 °C for 1 h, the plates were washed and incubated with 100 µL of horseradish peroxidase-conjugated recombinant protein A/G (1:10,000 dilution; Thermo Fisher Scientific, Rockford, IL, USA), previously reported to bind elephant IgG [42,43], at 37 °C for 1 h. Next, the peroxidase activity was determined by adding 100 µL of 3,3′,5,5′-tetramethylbenzidine (Invitrogen, Carlsbad, CA, USA), and the reaction was stopped by adding 100 µL of 1 N of HCl. The optical density (OD) was measured at a wavelength of 450 nm using an ELx808 ELISA microplate reader (Bio Tec Instruments, VT, USA). Positive and negative serum controls were confirmed using MAST Toxoreagent, a commercial LAT (Liverpool, UK), and were included in all plates. Serum samples were considered positive when the average OD > (OD mean [from the negative control sera] + 3 standard deviations from the negative control sera)].

### 4.6. Statistical Analyses

Data were analyzed using SPSS version 26.0 for Windows (IBM Corp., Armonk, NY, USA). The seroprevalence was calculated based on the ratio of positive results to the total number of tested animals. The association between *T. gondii* seropositive individuals and risk factors (age and sex) was analyzed using the chi-square test. A *p* value of <0.05 was considered statistically significant. The kappa values, specificity, sensitivity, and 95% confidence intervals were calculated using VassarStats (www.vassarstats.net (accessed on 25, October 2021)). The strength of agreement was graded according to the kappa values as fair (0.21–0.40), moderate (0.41–0.60), or substantial (0.61–0.80).

## Figures and Tables

**Table 1:** Seroprevalence of *T. gondii* antibody in Asian elephant sera by LAT, TLA-iELISA, and TgGRA8-iELISA.

Year of Sampling	Region	Province	No. of Elephants Tested	Seropositivity of *T. gondii*-Specific IgG Antibodies		
				LAT (Titer ≥ 1:64)	TLA	TgGRA8
2009	North	Mae Hong Sorn	6	4	4	1
	Western	Tak	44	23	28	18
	Central	Sukhothai	6	3	2	2
		Pathum Thani	4	1	1	1
	Northeast	Surin	1	1	0	0
	South	Chumphon	4	2	2	0
		Krabi	10	0	0	3
		Nakhon Si Thammarat	24	16	8	13
		Phangnga	4	3	4	3
		Patthalung	2	0	0	1
		Ranong	4	2	0	2
		Satun	1	0	0	0
		Surat Thani	12	2	1	3
		Trang	23	7	8	7
2010	Central	Ayutthaya	9	4	4	5
	South	Krabi	1	0	0	0
		Phuket	12	9	7	9
		Surat Thani	10	4	2	4
2011	East	Chonburi	5	2	3	2
	Northeast	Surin	6	1	1	2
	Western	Prachaup Kirikhan	2	0	0	2
2013	Northeast	Surin	1	0	0	1
	Western	Kanchanaburi	60	31	28	35
2020	Central	Nakhon Pathom	9	5	5	5
	Western	Kanchanaburi	8	1	1	0
		Total	268	121 (45.1%)	109 (40.7%)	119 (44.4%)

IgG: Immunoglobulin G; LAT: Latex agglutination test; TLA: *T. gondii* total lysate antigen; TgGRA8 = *T. gondii* dense granular antigen 8.

**Table 2:** Seroprevalence of *T. gondii* antibody (LAT; titer ≥ 64) in Asian elephant sera by sex and age-group.

Variable	Total Tested	LAT Positive (%)	OR (95% CI)	*p*-Value (<0.05)	Year of Sampling (No. of Positive Samples/Total Tested)
Sex					
Female	188	88 (46.8%)	Ref		2009 (38/90), 2010 (14/24), 2011 (2/13), 2013 (29/47), 2020 (5/14)
Male	80	33 (41.3%)	0.8 (0.5–1.4)	0.40	2009 (26/55), 2010 (3/8), 2011 (1/1), 2013 (2/13), 2020 (1/3)
Age					
1–20 years	62	42 (68.9%)	6.6 (2.9–15.4)	<0.001	2009 (12/21), 2010 (4/5), 2013 (25/34), 2020 (1/2)
21–40 years	96	48 (50%)	3.2 (1.5–6.8)	0.002	2009 (39/73), 2010 (1/1), 2013 (4/13), 2020 (4/9)
>40 years	50	12 (24%)	Ref		2009 (8/30), 2010 (1/1), 2013 (2/13), 2020 (1/6)
Unknown age	60	19 (31.7%)			

LAT: Latex agglutination test; Ref: Reference category; OR: odds ratio; CI: confidence interval.

**Table 3:** Comparison of TLA-iELISA and TgGRA8-iELISA to examine *T. gondii* antibodies in Asian elephants using LAT as a reference test.

		LAT			SE (%) (95%CI)	SP (%) (95%CI)	PPV (%) (95%CI)	NPV (%) (95%CI)	Kappa
		Positive	Negative	Total					
	Positive	95	14	109	78.5	90.5	87.2	83.6	0.69
TLA	Negative	26	133	159	(69.9–85.2)	(84.2–94.5)	(79–92.5)	(76.7–88.8)	
	Total	121	147	268					
	Positive	82	37	119	67.7	74.8	68.9	73.8	0.42
TgGRA8	Negative	39	110	149	(58.5–75.8)	(66.8–81.4)	(59.6–76.9)	(65.8–80.5)	
	Total	121	147	268					

SE: sensitivity, SP: specificity, PPV: positive predictive value, NPV: negative predictive value, CI: confidence interval, Kappa agreement value.

## Data Availability

Not applicable.

## References

[1] Montoya J.G., Liesenfeld O. (2004). Toxoplasmosis. Lancet.

[2] Stelzer S., Basso W., Benavides Silván J., Ortega-Mora L.M., Maksimov P., Gethmann J., Conraths F.J., Schares G. (2019). *Toxoplasma gondii* infection and toxoplasmosis in farm animals: Risk factors and economic impact. Food Waterborne Parasitol..

[3] Zulpo D.L., Sammi A.S., dos Santos J.R., Sass J.P., Martins T.A., Minutti A.F., Cardim S.T., de Barros L.D., Navarro I.T., Garcia J.L. (2018). *Toxoplasma gondii*: A study of oocyst re-shedding in domestic cats. Vet. Parasitol..

[4] Dubey J.P. (2009). History of the discovery of the life cycle of *Toxoplasma gondii*. Int. J. Parasitol..

[5] Maruyama S., Boonmar S., Morita Y., Sakai T., Tanaka S., Yamaguchi F., Kabeya H., Katsube Y. (2000). Seroprevalence of Bartonella henselae and *Toxoplasma gondii* among healthy individuals in Thailand. J. Vet. Med. Sci..

[6] Wanachiwanawin D., Sutthent R., Chokephaib U.K., Mahakittikun V., Ongrotchanakun J., Monkong N. (2001). *Toxoplasma gondii* antibodies in HIV and non-HIV infected Thai pregnant women. Asian Pac. J. Allergy Immunol..

[7] Nissapatorn V., Noor Azmi M.A., Cho S.M., Fong M.Y., Init I., Rohela M., Khairul Anuar A., Quek K.F., Latt H.M. (2003). Toxoplasmosis: Prevalence and risk factors. J. Obstet. Gynaecol. Res..

[8] Sukthana Y., Kaewkungwal J., Jantanavivat C., Lekkla A., Chiabchalard R., Aumarm W. (2003). *Toxoplasma gondii* antibody in Thai cats and their owners. Southeast Asian J. Trop. Med. Public Health.

[9] Jittapalapong S., Nimsupan B., Pinyopanuwat N., Chimnoi W., Kabeya H., Maruyama S. (2007). Seroprevalence of *Toxoplasma gondii* antibodies in stray cats and dogs in the Bangkok Metropolitan areas, Thailand. Vet. Parasitol..

[10] Jittapalapong S., Inpankaew T., Pinyopanuwat N., Chimnoi W., Kengradomkij C., Wongnarkpet S., Maruyama S., Lekkla A., Sukthana Y. (2010). Epidemiology of *Toxoplasma gondii* infection of stray cats in Bangkok, Thailand. Southeast Asian J. Trop. Med. Public Health.

[11] Udonsom R., Buddhirongawatr R., Nishikawa Y., Fereig R.M., Jirapattharasate C. (2021). *Toxoplasma gondii* prevalence and risk factors in owned domestic cats from Nakhon Pathom Province, Thailand. Vet. Integr. Sci..

[12] Huertas-López A., Sukhumavasi W., Álvarez-García G., Martínez-Subiela S., Cano-Terriza D., Almería S., Dubey J.P., García-Bocanegra I., Cerón J.J., Martínez-Carrasco C. (2021). Seroprevalence of *Toxoplasma gondii* in outdoor dogs and cats in Bangkok, Thailand. Parasitology.

[13] Inpankaew T., Pinyopanuwut N., Chimnoi W., Kengradomkit C., Sununta C., Zhang G., Nishikawa Y., Igarashi I., Xuan X., Jittapalapong S. (2010). Serodiagnosis of *Toxoplasma gondii* infection in dairy cows in Thailand. Transbound. Emerg. Dis..

[14] Wiengcharoen J., Nakthong C., Mitchaothai J., Udonsom R., Sukthana Y. (2012). Toxoplasmosis and neosporosis among beef cattle slaughtered for food in Western Thailand. Southeast Asian J. Trop. Med. Public Health.

[15] Udonsom R., Sukthana Y., Nishikawa Y., Fereig R.M., Jirapattharasate C. (2018). Current situation of *Neospora ganinum* and *Toxoplasma gondii* infection among beef cattle in Kanchanaburi, Ratchaburi and Nakhon Patom Provinces, Thailand. Thai J. Vet. Med..

[16] Saichua P., Jumnainsong A., Tantrawatpan C., Kiatsopit N., Kopolrat K., Suwannatrai A., Sithithaworn P. (2017). Seroprevalence of *Toxoplasma gondii* in free range chickens (Gallus domesticus) in Khon Kaen province, Thailand. Trop. Biomed..

[17] Jittapalapong S., Sangvaranond A., Pinyopanuwat N., Chimnoi W., Khachaeram W., Koizumi S., Maruyama S. (2005). Seroprevalence of *Toxoplasma gondii* infection in domestic goats in Satun Province, Thailand. Vet. Parasitol..

[18] Udonsom R., Supanta J., Tanglakmankhong O., Ngoenphisutsin K., Nishikawa Y., Fereig R.M., Jirapattharasate C. (2012). *Toxoplasma gondii* and *Neospora caninum* prevalence and risk factors on goat farms in Kanchanaburi province, Thailand. Vet. Integr. Sci..

[19] Thiangtum K., Nimsuphun B., Pinyopanuwat N., Chimnoi W., Tunwattana W., Tongthainan D., Jittapalapong S., Rukkwamsuk T., Maruyama S. (2006). Seroprevalence of *Toxoplasma gondii* in captive felids in Thailand. Vet. Parasitol..

[20] Buddhirongawatr R., Chaichoun K., Tungsudjai S., Udonsom R., Thompson A., Mahittikorn O., Dekumyoy P., Sukthana Y. (2016). Seroprevalence and phylogenetic analysis of *Toxoplasma gondii* from domestic Cats, captive wild felids, free-range wild felids and rats in certain regions of Thailand. Thai J. Vet. Med..

[21] Dubey J.P., Jones J.L. (2008). *Toxoplasma gondii* infection in humans and animals in the United States. Int. J. Parasitol..

[22] Khan A.H., Noordin R. (2020). Serological and molecular rapid diagnostic tests for Toxoplasma infection in humans and animals. Eur. J. Clin. Microbiol. Infect. Dis..

[23] Cai Y., Wang Z., Li J., Li N., Wei F., Liu Q. (2015). Evaluation of an indirect ELISA using recombinant granule antigen GRA7 for serodiagnosis of *Toxoplasma gondii* infection in cats. J. Parasitol..

[24] Wang Z., Ge W., Huang S.Y., Li J., Zhu X.Q., Liu Q. (2014). Evaluation of recombinant granule antigens GRA1 and GRA7 for serodiagnosis of *Toxoplasma gondii* infection in dogs. BMC Vet. Res..

[25] Abdelbaset A.E., Alhasan H., Salman D., Karram M.H., Ellah Rushdi M.A., Xuenan X., Igarashi M. (2017). Evaluation of recombinant antigens in combination and single formula for diagnosis of feline toxoplasmosis. Exp. Parasitol..

[26] Ferra B., Holec-Gąsior L., Kur J. (2015). Serodiagnosis of *Toxoplasma gondii* infection in farm animals (horses, swine, and sheep) by enzyme-linked immunosorbent assay using chimeric antigens. Parasitol. Int..

[27] Ferra B., Holec-Gąsior L., Grąźlewska W. (2020). *Toxoplasma gondii* recombinant antigens in the serodiagnosis of toxoplasmosis in domestic and farm Animals. Animals.

[28] Wiengcharoen J., Nokkaew W., Prasithpon S., Prasomtong P., Sukthana Y. (2012). *Neospora caninum* and *Toxoplasma gondii* antibodies in captive Elephants (*Elephaus Maximus Indicus*) in Kanchanaburi Province. Thai J. Vet. Med..

[29] Tuntasuvan D., Mohkaew K., Dubey J.P. (2001). Seroprevalence of *Toxoplasma gondii* in Elephants (*Elephus maximus indicus*) in Thailand. Parasitology.

[30] Bansiddhi P., Brown J.L., Thitaram C. (2020). Welfare Assessment and Activities of Captive Elephants in Thailand. Animals.

[31] Shahiduzzaman M., Islam R., Khatun M.M., Batanova T.A., Kitoh K., Takashima Y. (2011). *Toxoplasma gondii* seroprevalence in domestic animals and humans in Mymensingh District, Bangladesh. J. Vet. Med. Sci..

[32] Matsuo K., Kamai R., Uetsu H., Goto H., Takashima Y., Nagamune K. (2014). Seroprevalence of *Toxoplasma gondii* infection in cattle, horses, pigs and chickens in Japan. Parasitol. Int..

[33] Bártová E., Lukášová R., Vodička R., Váhala J., Pavlačík L., Budíková M., Sedlák K. (2018). Epizootological study on *Toxoplasma gondii* in zoo animals in the Czech Republic. Acta Trop..

[34] Obwaller A., Hassl A., Picher O., Aspock H. (1995). An enzyme-linked immunosorbent assay with whole trophozoites of *Toxoplasma gondii* from serum-free tissue culture for detection of specific antibodies. Parasitol. Res..

[35] Babaie J., Miri M., Sadeghiani G., Zare M., Khalili G., Golkar M. (2011). Expression and Single-step Purification of GRA8 Antigen of *Toxoplasma gondii* in *Escherichia coli*. Avicenna J. Med. Biotechnol..

[36] Jirapattharasate C., Udonsom R., Prachasuphap A., Jongpitisub K., Dhepakson P. (2021). Development and evaluation of recombinant GRA8 protein for the serodiagnosis of *Toxoplasma gondii* infection in goats. BMC Vet. Res..

[37] Koethe M., Pott S., Ludewig M., Bangoura B., Zöller B., Daugschies A., Tenter A.M., Spekker K., Bittame A., Mercier C. (2011). Prevalence of specific IgG-antibodies against *Toxoplasma gondii* in domestic turkeys determined by kinetic ELISA based on recombinant GRA7 and GRA8. Vet. Parasitol..

[38] Song Y., Zhao Y., Pan K., Shen B., Fang R., Hu M., Zhao J., Zhou Y. (2021). Characterization and evaluation of a recombinant multiepitope peptide antigen MAG in the serological diagnosis of *Toxoplasma gondii* infection in pigs. Parasites Vectors.

[39] Cuhadar S., Koseoglu M., Atay A., Dirican A. (2013). The effect of storage time and freeze-thaw cycles on the stability of serum samples. Biochem. Med..

[40] Kempf J., Melliger R.H., Reusch C.E., Kook P.H. (2018). Effects of storage conditions and duration on cobalamin concentration in serum samples from cats and dogs. J. Am. Vet. Med. Assoc..

[41] Dard C., Bailly S., Drouet T., Fricker-Hidalgo H., Brenier-Pinchart M.P., Pelloux H. (2017). Long-term sera storage does not significantly modify the interpretation of toxoplasmosis serologies. J. Microbiol. Methods.

[42] Hoornweg T.E., Schaftenaar W., Maurer G., van den Doel P.B., Molenaar F.M., Chamouard-Galante A., Vercammen F., Rutten V., de Haan C. (2021). Elephant endotheliotropic Herpesvirus is omnipresent in elephants in European zoos and an Asian elephant range country. Viruses.

[43] Paungpin W., Wiriyarat W., Chaichoun K., Tiyanun E., Sangkachai N., Changsom D., Poltep K., Ratanakorn P., Puthavathana P. (2017). Serosurveillance for pandemic influenza A (H1N1) 2009 virus infection in domestic elephants, Thailand. PLoS ONE.

